# Hypertension Call to Action: Will We Respond to the Call With Action?

**DOI:** 10.1093/ajh/hpab191

**Published:** 2022-03-08

**Authors:** Stephanie K Mayfield, Kathryn Foti, Andrew E Moran, Drew E Blakeman, Thomas R Frieden

**Affiliations:** 1 Resolve to Save Lives, an initiative of Vital Strategies, New York, New York, USA; 2 Department of Epidemiology, Johns Hopkins Bloomberg School of Public Health, Baltimore, Maryland, USA; 3 Columbia University Irving Medical Center, New York, New York, USA

The COVID-19 pandemic has so far resulted in more than 800,000 deaths in the United States alone and presented profound challenges to every sector of society, including public health and health care. One such challenge is addressing the impact of delayed or avoided non-COVID-related health care by 41% of US adults in the context of high prevalence of chronic diseases and longstanding health disparities.^1^

In the United States, approximately one third of adults have hypertension (blood pressure ≥140/90 mm Hg or taking antihypertensive medication). Even before the pandemic, decades of national progress controlling hypertension had reversed; less than half of all adults with hypertension now have blood pressure controlled to <140/90 mm Hg.^2^ Furthermore, hypertension prevalence is higher among non-Hispanic Black adults than non-Hispanic White adults, and among those with hypertension the proportion with controlled blood pressure is lower, resulting in stark and persistent disparities. Between 2015 and 2018, only 41.5% of non-Hispanic Black adults with hypertension had controlled blood pressure, compared with 48.2% of non-Hispanic White adults.^2^ Blood pressure control was also higher in adults with private insurance (48.2%) or Medicare (53.4%) compared with those without health insurance (24.2%), as well as among those who had a health care visit in the past year (49.1%) compared with those who did not (8%).^2^

In 2017, the American College of Cardiology and American Heart Association published updated blood pressure guidelines that redefined hypertension as blood pressure >130/80 mm Hg or taking antihypertensive medication. By this definition, hypertension prevalence among US adults is nearly 50%; of those, only 1 in 5 has blood pressure controlled to <130/80 mm Hg.^2^ Hypertension and the COVID-19 pandemic have each resulted in hundreds of thousands of deaths this year alone and have also exacerbated long-existing health inequities. However, far too little attention is paid to the public health crisis of hypertension because it is not novel and rarely in the headlines.

We understand the risk factors that contribute to hypertension and have long had low-cost, effective treatments to control it.^3^ So, why do we continue losing so many lives to hypertension? The simple answer is that we fail to implement proven prevention interventions and treat hypertension effectively, in large part because the financial incentives of our health care system are not aligned with the goal of controlling blood pressure. Small tweaks to our “business as usual” approach to prevent and control hypertension are bound to continue to fall short, as they have for decades. Only a comprehensive approach—a nationally driven, whole-of-government and whole-of-community approach with a specific focus on financial incentives—will turn the tide. This is the approach recommended in the 2020 US Surgeon General’s Call to Action to Control Hypertension, which sets 3 overarching goals:

## MAKE HYPERTENSION CONTROL A NATIONAL PRIORITY

To make hypertension control a national priority, specific policy initiatives must be pursued at the federal level. First, the federal government can improve nutrition by promoting a whole-of-government approach that shifts the national food supply to a healthier profile. Elevated sodium intake is associated with raised blood pressure, hypertension, and cardiovascular disease. Since 70% of sodium in diets comes from packaged, processed, and restaurant foods, it is appropriate for government agencies to use regulatory authority to reduce sodium content in foods. The US adult population consumes on average 50% more sodium than the daily recommended amount (a maximum 2,300 mg for people aged 14 years and older). Of children aged 2–13 years, 95% exceed recommended dietary sodium limits for their age groups.^4^

Recent action by the US Food & Drug Administration (FDA) to implement voluntary sodium reduction guidelines for industry aim to reduce daily sodium intake by 12%, from 3,400 to 3,000 mg, over the next 2.5 years. The FDA estimates that, if industry adheres to this guidance, tens of thousands of lives and billions of dollars in health care costs will be saved in coming years. However, because sodium intake would still exceed levels recommended for chronic disease risk reduction, the FDA plans further incremental reductions over coming years.

## ENSURE THAT THE PLACES WHERE PEOPLE LIVE, LEARN, WORK, AND PLAY SUPPORT HYPERTENSION PREVENTION STRATEGIES AND CONTROL

Obesity is a major risk factor for essential hypertension, and its prevalence continues to increase.^5^ Sixteen states had adult obesity prevalence at or above 35% in 2020, up from 12 states in 2019. Obesity prevalence in US children ages 2–19 is nearly 20%, increasing their risk for developing chronic diseases later in life. With the support of government at all levels, communities should have access to healthy school meals that are free; assured funding for Supplemental Nutrition Assistance Program benefits and more healthful choices in that program; expansion of the Special Supplemental Nutrition Program for Women, Infants and Children; and increased availability of heart-healthy meals distributed through senior services programs.

Employers can provide a supportive environment for healthy nutrition in worksite cafeterias and vending machines; offer worksite blood pressure monitoring, physical activity, and tobacco use cessation programs; and champion a “know your numbers” culture where ubiquitous accessibility to blood pressure monitors empowers individuals to take charge of their health and know if they are in a healthy blood pressure range. Governments should also promote policies for community design that increase opportunities for physical activity for all people by creating safe activity-friendly routes to everyday destinations.

Community organizations and public–private partnerships can support programs to address social determinants of health and improve equity. African Americans are at increased risk of hypertension compared with other racial and ethnic groups, and chronic stress from structural and interpersonal racism likely contributes to increased risk. In the Jackson Heart Study, higher stress from lifetime discrimination was associated with higher hypertension risk after adjusting for risk factors such as smoking, obesity, and lack of physical activity.^6^ Community organizations, in collaboration with public–private partnerships, can actively address structural racism, including through improved data collection and development of effective interventions that address not only individual behaviors, but also the downstream effects of racism on social determinants of health.

## OPTIMIZE PATIENT CARE FOR HYPERTENSION

Quality hypertension care starts with health care access for all, and improved services for those with access. One study involving Federally Qualified Health Centers noted that Medicaid expansion was associated with modest improvements in hypertension control and sustained increases in insurance coverage.^7^ The federal government can support states to increase the number of people covered by insurance while reducing restrictions on coverage; reduce or remove co-pays for core antihypertensive medications; provide access to the most efficacious medications with the simplest treatment regimens; and remove obstacles that delay or prevent care such as preauthorization for preventive treatments.

Team-based care using outpatient multidisciplinary teams improves hypertension control^8^ and has the potential to reduce disparities in blood pressure control. Community-based models can leverage trusted relationships with community partners to deliver hypertension screening and treatment services where people live or work. The Centers for Medicare & Medicaid Services should support innovative payment models for concerted hypertension control programs including community-based service delivery, team-based care, and blood pressure self-monitoring combined with clinical support, reimbursing these services through insurance programs.^9^ Public and private payors should continue to support telehealth as a sustainable access point to hypertension care, especially in rural communities that lack primary care clinics.

Ultimately, however, these important practice improvements in hypertension care are unlikely to occur and be sustained unless financial incentives change. In the US health care system, failure to elevate prevention over sick care increases health system revenues and profits because mismatched economic incentives favor compensation for acute care over preventive care and specialty care over primary care. Systems with integrated primary and hospital care and insurance coverage, such as Kaiser Permanente, have financial incentives to improve hypertension treatment and are much more likely to adopt specific treatment protocols; buy the best and safest medications; create coordinated multidisciplinary health care teams to support patients; remove financial and other barriers that discourage patients from continuing treatment; and establish accurate, real-time information systems to improve quality rapidly.

Over the past 40 years, the United States has gone from having a near-average life expectancy for upper income countries and near-average per capita health care costs to being a negative outlier.^10^ Compensation models that make primary care the center of our health care system and pay for effective, high-quality, high-value care can prevent disease and reduce total health care costs.

Public health agencies can play a pivotal role at the community level by promoting hypertension awareness and education and leading multisector coalitions. They can also, in collaboration with health care delivery systems, collect and analyze data to map communities at highest risk of uncontrolled blood pressure and focus multisector interventions to reduce their high burden of hypertension-related disease. In partnership with public and private sectors, public health systems can guide these interventions and help mobilize a cadre of community health workers to improve care and help reduce health disparities. Communities with a high hypertension burden should have access to additional targeted funding through various sources such as public health insurance programs and public employee health plans to cover the costs of proven hypertension control interventions, optimally tailoring them to local contexts.

In conclusion, hypertension is a leading preventable risk factor for death in the United States. The COVID-19 pandemic has highlighted the importance of addressing the social determinants of health and optimizing patient-centered care to improve health outcomes and reduce racial and ethnic disparities. These same approaches are necessary to improve prevention and control of hypertension. The time is now to respond to the Surgeon General’s Call to Action to Control Hypertension—with action.

## Data Availability

All data are derived from public repositories or published material and can be accessed where cited.
